# Invasion Biology Meets Parasitology: A Case Study of Parasite Spill-Back with Egyptian *Fasciola gigantica* in the Invasive Snail *Pseudosuccinea columella*

**DOI:** 10.1371/journal.pone.0088537

**Published:** 2014-02-11

**Authors:** Daniel S. Grabner, Faten A. M. M. Mohamed, Milen Nachev, Eman M. H. Méabed, Abdel Hameed A. Sabry, Bernd Sures

**Affiliations:** 1 Aquatic Ecology and Centre for Water and Environmental Research, University of Duisburg-Essen, Essen, Germany; 2 Parasitology Department, Faculty of Medicine, Fayoum University, Fayoum, Egypt; Macquarie University, Australia

## Abstract

The liver fluke *Fasciola gigantica* is a trematode parasite of ruminants and humans that occurs naturally in Africa and Asia. Cases of human fascioliasis, attributable at least in part to *F. gigantica*, are significantly increasing in the last decades. The introduced snail species *Galba truncatula* was already identified to be an important intermediate host for this parasite and the efficient invader *Pseudosuccinea columella* is another suspect in this case. Therefore, we investigated snails collected in irrigation canals in Fayoum governorate in Egypt for prevalence of trematodes with focus on *P. columella* and its role for the transmission of *F. gigantica*. Species were identified morphologically and by partial sequencing of the cytochrome oxidase subunit I gene (*COI*). Among all 689 snails found at the 21 sampling sites, *P. columella* was the most abundant snail with 296 individuals (42.96%) and it was also the most dominant species at 10 sites. It was not found at 8 sites. Molecular detection by PCR and sequencing of the ITS1-5.8S-ITS2 region of the ribosomal DNA (rDNA) revealed infections with *F. gigantica* (3.38%), *Echinostoma caproni* (2.36%) and another echinostome (7.09%) that could not be identified further according to its sequence. No dependency of snail size and trematode infection was found. Both high abundance of *P. columella* in the Fayoum irrigation system and common infection with *F. gigantica* might be a case of parasite spill-back (increased prevalence in local final hosts due to highly susceptible introduced intermediate host species) from the introduced *P. columella* to the human population, explaining at least partly the observed increase of reported fascioliasis-cases in Egypt. *Eichhornia crassipes*, the invasive water hyacinth, which covers huge areas of the irrigation canals, offers safe refuges for the amphibious *P. columella* during molluscicide application. As a consequence, this snail dominates snail communities and efficiently transmits *F. gigantica*.

## Introduction

The liver fluke *Fasciola gigantica* is a trematode parasite native to Africa and Asia and infects ruminants, but also humans as final hosts. Adult *F. gigantica* live in the bile ducts of the liver, where they can reach a length of up to 76 mm. Infection with this parasite can cause severe disease symptoms referred to as fascioliasis [1]. Intermediate hosts are various lymnaeid snails [2], inside of which the parasite proliferates asexually and produces free swimming cercariae that will attach to submerged surfaces, mostly plants. These cercariae develop into encysted and durable metacercariae that are transmitted when the final host ingests the metacercariae together with plants, or by consumption of water contaminated with metacercariae [3]. Due to the durability of the metacercariae, transmission can also be mediated by ingestion of terrestrial plants and crops that were submerged in water containing infected snails for a couple of weeks [1], [4], which is a common irrigation technique in Fayoum area. Especially in Egypt, fascioliasis is an increasing problem, reaching prevalences in animals of sometimes more than 50% and up to 19% in humans [5]–[7]. According to estimations of the World Health Organization [8], at least 830,000 people are infected with either the introduced *Fasciola hepatica* or *F. gigantica* in the Nile delta. Presence of both of these closely related species has been confirmed for Egypt [1], [9], [10], but they are usually not distinguished diagnostically [11].

The natural first intermediate host of *F. gigantica* in Egypt is the snail *Radix natalensis*, but the trematode was also found commonly in the introduced species *Galba truncatula* [12]. Additionally, single cases of *F. gigantica* infections were reported from *Biomphalaria alexandrina* and *Pseudosuccinea columella* in Egypt [13]–[15]. For the latter species, release of *F. gigantica* cercariae was proven in laboratory infections [16]. The highly invasive lymnaeid snail *P. columella* was introduced from North America to many countries worldwide and was reported from Africa for the first time in the middle of the 20^th^ century, where it is now widely distributed [17]–[20]. *P. columella* is well known as a suitable host for *F. hepatica* [12], but its importance for the maintenance of the natural life cycle of *F. gigantica* and the transmission to humans is uncertain.

According to the parasite spill back hypothesis, invasive species are colonized by local parasites. If the invader becomes abundant and the parasite can develop successfully, a high number of transmitting stages will develop and increase local parasite abundance and prevalence [21]. In this way, free living invasive species may help native parasites to increase their population size and extend their distribution range [22]. *P. columella* is a very efficient invader and became an important snail host for *F. hepatica* in many countries [12]. We hypothesize that this might be the case for the *P. columella*/*F. gigantica*–system as well. As a result, infected *P. columella* might be responsible for the observed increase in infection intensity and prevalence in livestock and in the human population in Egypt. Therefore, we investigated the trematode species occurring in the invasive snail *P. columella*, collected from irrigation channels in the Fayoum governorate where cases of fascioliasis are commonly reported [23], [24]. We assessed the potential of this invasive species as a host for trematodes, especially for *Fasciola* spp. to estimate the effect of *P. columella* for the spread of fascioliasis in the area and found evidence for a possible spill-back effect on animals and the human population.

## Materials and Methods

### Snail sampling

Snails were collected from July to September 2012, 2 month after the last molluscicide treatment, from water plants (mainly water hyacinths *Eichhornia crassipes*) and with dip nets in irrigation channels at 21 different sites in Fayoum governorate (surrounding Markaz El-Fayoum, Itsa, and Ibshway cities), Egypt. Samples were taken with permission of the local farmers owning the land adjacent to the irrigation channels. No endangered or protected species were sampled. Sampled snails were fixed in 99% ethanol for molecular analysis. Collected specimens from the different sampling sites were identified morphologically according to the key of Brown [19] and by molecular biology. After identification, *P. columella* individuals were measured (shell length) and crushed to check visually for trematode infections. Samples of the soft tissue (for molecular analysis of parasites) and the foot muscle (for molecular species identification of all snails; presumed to be free of parasites) were taken and frozen at −20°C for molecular analyses.

### Molecular analyses

Snail tissue samples were homogenized in 1.5 ml reaction tubes with micropestles (Eppendorf) and DNA was extracted with a JETQUICK DNA Clean-Up Spin Kit (Genomed) according to manufacturer's instructions. Molecular species identification of the snails was done by sequencing of the Folmer-region of the cytochrome oxidase subunit 1 (*COI*) with the primers LCO1490 and HC02198 [25] (about 700 bp). At least two individuals of each species were sequenced to confirm morphological identification.

For molecular detection of trematode infections in the soft tissue of *P. columella*, the universal trematode primers Trem1 F/Trem1 R were designed that amplify a short part of the internal transcribed spacer 2 (ITS2) and the beginning of the 28S ribosomal DNA (rDNA) (about 200 bp). For sequencing, an additional PCR with the primers Trem2 F (end of 18S rDNA) and Trem1 R was conducted to obtain a longer sequence, including almost the whole internal transcribed spacer 1 (ITS1) – 5.8S rDNA– ITS2 region of the ribosomal DNA (about 1300 bp). The primer pair Fasc-ITS1 F/R was designed for specific amplification of a 716 bp ITS1-segment of both *F. gigantica* and *F. hepatica*, to distinguish *Fasciola* spp. from host and infections with other trematodes. Sequences and additional information for primers designed for the present study are given in table 1. One 20 µl PCR reaction mix contained 4 µl of 5× Crimson Taq buffer (New England Biolabs), 0.2 mM dNTP mix (New England Biolabs), 0.5 µM of each primer 0.5 U Crimson Taq (New England Biolabs) and 1 µl template DNA. The mix was topped up to 20 µl with PCR grade water. The DNA was amplified by a Labcycler (SensoQuest) under the following conditions: initial denaturation at 95°C for 5 min, 40 cycles of 95°C for 30 s, annealing (temperatures see table 1) for 30 s and elongation at 72°C for 30 s followed by a final elongation of 72°C for 5 min.

**Table 1:** Sequences of primers designed for the present study and annealing temperatures.

Name	Sequence	Target region	Annealing Temp.	Approximate length of product
Trem1 F	TAG CCT YGG ATC AGW CGT GA	ITS2	54°C	200 bp with Trem1 R
Trem2 F	CAA GTC ATA AGC TTG CGC TGA	18S rDNA	54°C	1300 bp with Trem1 R
Trem1 R	ACC YAA ACA CCA CAT TGC CTA	28S rDNA	54°C	
Fasc ITS1 F	TCT ACT CTT ACA CAA GCG ATA CAC	ITS1	55°C	716 bp
Fasc ITS1 R	GGC TTT CTG CCA AGA CAA G	ITS1		

Sensitivity of the Trem2 F/Trem1 R primer pair was lower and not all samples were amplified successfully with the Crimson Taq, although tested positive before with Trem1 F/Trem1 R primers. Therefore, the more robust Phire Animal Tissue Direct PCR Kit (Thermo Scientific) was used for amplification with those primers. Reactions contained 10 µl of 2× Phire PCR Buffer, 0.5 µM of each primer, 0.4 µl Phire Hot Start II DNA Polymerase and 1 µl DNA. Water was added to 20 µl. PCR conditions were 98°C for 5 min, 40 cycles of 98°C for 10 s, annealing at 54°C for 10 s, elongation at 72°C for 20 s and a final elongation at 72°C for 1 min. PCR conditions for the LCO1490/HC02198 primers were as described in Folmer et al. [25]. PCR products were checked by standard agarose gel electrophoresis, purified with a JETQUICK PCR Product Purification Spin Kit (Genomed) according to manufacturer's instructions and sent for sequencing (GATC). Sequences were checked for homology with database entries by BLAST searches (http://blast.ncbi.nlm.nih.gov/Blast.cgi).

### Statistics

Mean prevalences and confidence intervals of parasite infections were calculated with the program Quantitative Parasitology v.3.0 [26]. Dependency of snail size and infection was analyzed by logistic regression using R v.3.0.1 [27].

## Results

### Snails

In total, 9 different snail species were identified morphologically and genetically. The sequences of different isolates of each species were identical, so one representative *COI*-sequence of each species was deposited in GenBank. Sorted by their abundance, the following species were found: *Pseudosuccinea columella* (accession no. KF412765), *Physa heterostropha* (accession no. KF412768), *Cleopatra bulimoides* (accession no. KF412769), *Bulinus truncatus* (accession no. KF412767), *Melanoides tuberculata* (accession no. KF412770), *Biomphalaria alexandrina* (accession no. KF412766), *Succinea* sp. (accession no. KF412772), *Bellamya* sp. (accession no. KF412773) and *Theodoxus anatolicus* (accession no. KF412771). The five *COI* sequences obtained from *P. columella* individuals were between 97%–100% similar to the different isolates of *P. columella* in GenBank.

**Figure 1:**
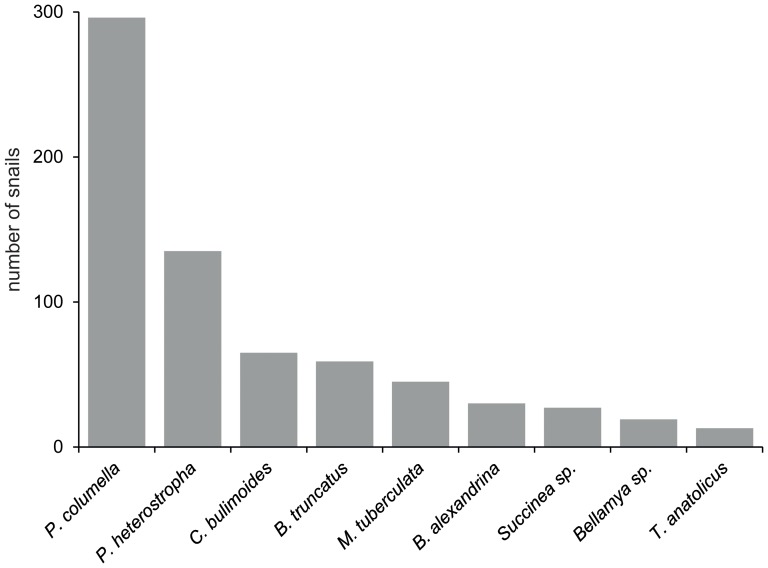
Total numbers of the snail species found at all sites.

Numbers of snails and species diversity varied greatly between sampling sites. The highest number of snails was found at Disya with 89 individuals (83 *P. heterostropha* and six *B. truncates*), only two snails of one species (*C. bulimoides*) were found at the Qalamshah village site. The most diverse snail community was present at the Zawyet El-Karadsah-site with 6 different snail species, while only a single snail species was found at two sites (*P. heterostropha* in El-Girb village and *C. bulimoides* in Qalamshah village). *P. columella* was not only the most abundant snail in total numbers (296 of 689, 42.96%; see fig. 1), but it was also the most dominant species at 10 sites and the second most dominant at one site. It was not found at eight of the 21 sampling sites. The second and third most abundant snails were *P. heterostropha* (19.59%) and *C. bulimoides* (9.43%), respectively (fig. 1). The latter species was found mostly in the locality of Izbat Ashur and Hawwarat Al-Maqta. Proportions of the other snails found in the area were 8.56% for *B. truncatus*, 6.53% for *M. tuberculata*, 4.35% for *B. alexandrina*, 3.92% for *Succinea* sp., 2.76% for *Bellamya* sp. and 1.89% for *Theodoxus anatolicus*. Detailed results on the number of snails and snail species at each site are shown in table 2. Size range of *P. columella* individuals varied between 0.15 and 1.18 cm. Mean and median size were 0.54 and 0.50 cm, respectively.

**Table 2:** Number and species of snails collected at the different sites. Sorted by number of indidiviuals per site.

Sampling sites	*Pseudo-succinea columella*	*Physa hetero-stropha*	*Cleopatra buli-moides*	*Bulinus truncatus*	*Melano-ides tuber-culata*	*Biom-phalaria alex-andrina*	*Succinea sp.*	*Bellamya sp.*	*Theo-doxus ana-tolicus*	No. of individuals (no. of species)
Disya village		83		6						89 (2)
Itsa (Bahr El Ghaba)	68						8			76 (2)
Al Amiriyah village	56						8			64 (2)
Izbat Ashur			38		10			2		50 (3)
Zawyet El-Karadsah	1	8	4	15			2	14		44 (6)
Hawwarat Al-Maqta		4	11	19			8		2	44 (5)
Sayyidna Al-Khidr village	42						1			43 (2)
Izbat El-Bank	14	7	6		4					31 (4)
Ahmed Afandi village	24	2	2		2					30 (4)
El-Misharrak village	26	4								30 (2)
Abu Ish	14					12		1		27 (3)
Itsa (Bahr Arus)	18				11					29 (2)
Izbat Hamada Dahman		7			3	3			11	24 (4)
Tutun village	8					13				21 (2)
El-Atamna Itsa village		5	2	11						18 (3)
Al Hadeer village	9	1			8					18 (3)
Izbat Ezbat El Eslah	2	1		8	4			2		17 (5)
El–Khawagat village	14				2					16 (2)
El–Girb village		13								13 (1)
Hanna Habib village					1	2				5 (2)
Qalamshah village			2							2 (1)
**Total no.**	296	135	65	59	45	30	27	19	13	689 (9)
**Proportion of total no.**	42.96%	19.59%	9.43%	8.56%	6.53%	4.35%	3.92%	2.76%	1.89%	

### Trematodes

**Figure 2:**
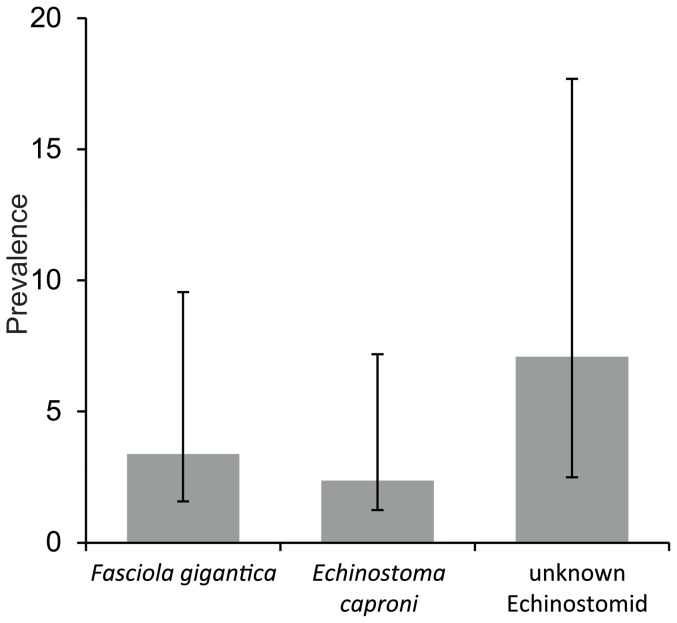
Total prevalence of the trematodes detected in *P. columella*. Error bars: 95% CI.

No trematode stages were found in *P. columella* specimens by visual inspection, as ethanol fixation made differentiation between host and parasite tissue impossible. By PCR with the Trem1 F/Trem1 R primers, 38 of the 296 *P. columella* specimens collected in total at all sampling sites were positive for trematode infection (12.84% [confidence interval (CI) 9.42–17.19%]). Sequencing of the ITS1-5.8S-ITS2 rDNA regions (with the Trem2 F/Trem1 R primers) revealed that 7 (2.36% [CI 1.12–4.83%]) of those snails were infected with *Echinostoma caproni* (accession no. KF425322) according to 98% sequence identity with AJ564382 (isolate from Cairo). *F. gigantica* was detected in 10 *P. columella* individuals (3.38% [CI 1.81–6.18%]) by PCR with Fasc ITS1 F/R primers and sequencing with Trem2 F/Trem1 R (accession no. KF425321; distinguished from *F. hepatica* according to variable positions listed in Mas-Coma et al. 2009). An infection with an unknown echinostome trematode was found in 21 *P. columella* individuals (7.09% [CI 4.60–10.60%]). The closest match for this sequence in the GenBank was the echinostome *Philophthalmus lucipetus* with only 85% similarity, therefore a more detailed identification was not possible. Figure 2 illustrates the overall prevalences of the three trematodes found. The highest prevalence for trematode infections was found in *P. columella* from El-Misharrak site, where 9 of 26 snails (34.62%) were infected, while the lowest infection rate was detected in Itsa (Bahr Arus) with one infected snail among 18 (5.56%). *E. caproni* was found at three and the unknown echinostomid at 8 out of 10 sites. *F. gigantica* infected snails were present at 5 of the 21 sampling sites with a prevalence of up to 11.11% (Al Hadeer village), but with only one infected snail out of nine. The lowest prevalence of *F. gigantica* was detected in Sayyidna Al-Khidr village with 4.76% (2 out of 42 *P. columella*). At three sites, no trematode infection was detected in *P. columella*, but in these cases snail numbers were low (8 in Tutun village, 2 in Izbat Ezbat El-Eslah El-Zraei and 1 in Zawyet El-Karadsah village). The infection rates per sampling site are summarized in table 3.

**Table 3:** Number of infected *P. columella* and prevalences for each trematode and site (total no. of tested snails given in brackets).

Site	*Fasciola gigantica*	*Echinostoma caproni*	unknown Echinostomid	all trematodes
El-Misharrak village	2 (26)/7.69%	2 (26)/7.69%	5 (26)/19.23%	9 (26)/34.62%
Sayyidna Al-Khidr village	2 (42)/4.76%	2 (42)/4.76%	3 (42)/7.14%	7 (42)/16.67%
Itsa (Bahr El Ghaba)		3 (68)/4.41%	7 (68)/10.29%	10 (68)/14.71%
El–Khawagat village			2 (14)/14.29%	2 (14)/14.29%
Al Hadeer village	1 (9)/11.11%			1 (9)/11.11%
Ahmed Afandi village	2 (24)/8.33%			2 (24)/8.33%
Al Amiriyah village	3 (56)/5.36%		1 (56)/1.79%	4 (56)/7.14%
Abu Ish			1 (14)/7.14%	1 (14)/7.14%
Izbat El-Bank			1 (14)/7.14%	1 (14)/7.14%
Itsa (Bahr Arus)			1 (18)/5.56%	1 (18)/5.56%
Total no. and mean prevalence with 95% CI	10 (296) 3.38% [1.81–6.18%]	7 (296) 2.36% [1.12–4.83%]	21 (296) 7.09% [4.60–10.60%]	38 (296) 12.84% [9.42–17.19%]

Results sorted in decreasing order by prevalence of all trematodes.

Although trematode infected snails were slightly smaller (mean 0.48 cm, median 0.46 cm) than uninfected snails (mean 0.54 cm, median 0.50 cm), no significant relationship between snail size and trematode infection was found (p = 0.26). *P. columella* infected with *F. gigantica* were on average 0.40 cm long (median 0.46 cm). Also for this parasite, no significant relationship between infection and size was found (p = 0.99).

## Discussion

The results of the present study show that the invasive snail *P. columella* is found frequently in irrigation channels in the Fayoum governorate (at 61.90% of sites investigated in the present study) and it even turned out to be the most abundant snail species at most sites. Additionally, our findings imply that *P. columella* probably became one of the major snail intermediate host species for *F. gigantica*. Although *F. gigantica* infections in *P. columella* have been reported before [14], this is the first study to provide molecular data for this host-parasite relationship. Possibly, the spread of *P. columella* as an additional intermediate host explains partly the increase of human fascioliasis cases in Egypt in the last 50 years [28], as well as the occurrence of hyperendemic outbreaks in the Nile delta [3], [6]. Both *Fasciola* species have been identified from parts of Egypt [9], [29], [30], but until now, no information was available for Fayoum governorate. Only *F. gigantica* was found in the irrigation system of the Fayoum oasis in the present study, indicating that this parasite is the major cause of fascioliasis-cases reported in humans [23] and animals [7], [24] in that area. The host snail, *P. columella*, is well known to be a suitable host for *F. hepatica* [12], but apparently it can maintain the life cycle of *F. gigantica* as well. Surprisingly, *Radix natalensis*, the indigenous host for *F. gigantica* was not found at all within the present study. This might be explained by seasonal peaks in abundance of the two species reported by Ahmed & Ramzy [14]. These authors observed that *P. columella* was predominant in autumn and *R. natalensis* from December to February. If this is the case in the area investigated in the present study, it might come to an increased infection pressure due to the presence of infected intermediate hosts throughout the year. Another reason for the absence of *R. natalensis* in the samples of the present study might also be the high requirements of this species on water quality and oxygen levels [14], [31] that can be limiting in the eutrophic irrigation channels, especially at high temperature. Compared to *P. columella*, *R. natalensis* might also be more sensitive to the molluscicide treatment in the channels, which gives the invader an additional selective advantage. In Brazil, *P. columella* was reported to be the only available host snail in some areas where *F. hepatica*-infections were reported [12], therefore this snail might also be able to maintain the life cycle of *F. gigantica* in Egypt. During a parasitological examination of lymnaid snails in Dakahlia governorate (sampling date was not reported), El-Shazly et al. [32] found mainly *R. natalensis* (68.4%) as well as *G. truncatula* (16.0%), but only few *P. columella* (3.4%). Also, no infection with *Fasciola* sp. was detected in *P. columella* in their study, indicating that *F. gigantica* is transmitted mainly by the natural intermediate host if it is present. According to previous experimental studies, *P. columella* is less susceptible to Cuban *F. hepatica* and produced lower numbers of rediae than the indigenous *Galba cubensis* [33]. This indicates that *P. columella* might not be a relevant host for *F. hepatica* (and possibly *F. gigantica* as well), if the natural snail host is present.

Several studies reported a rather high prevalence of liver flukes in their snail hosts. Caron et al. [34] found 6.25% of *G. truncatula* infected with *Fasciola* sp. in Algeria. In Dakahlia governorate, similar prevalences were reported in *R. natalensis* (5.50%) and *G. truncatula* (3.10%) [32]. Prevalences of *F. gigantica* observed in the present study (mean 3.83%) were within the range that was reported from *P. columella* in a previous study in Giza governorate [14], although *F. gigantica* infected snails in the present study were smaller (average of 0.40 cm) than reported by Ahmed & Ramzy [14], who noticed that most infected snails were larger than 1 cm. This might be due to the generally rather small size of snails in the present study with only a few individuals larger than 1 cm. Apparently, the infection is not restricted to large snails and can also occur in populations where only smaller snails are present.

One important factor that might lead to increased abundance of *P. columella*, but also other amphibious snails, is the water hyacinth (*Eichhornia crassipes*) that is present in most surface waters in Egypt. Like *P. columella*, *E. crassipes* is an invasive species in Egypt. It was introduced in Africa by the end of the 19^th^ century and spread throughout the continent [35]. According to recent estimations, the total area infested with water hyacinths in Egypt is as large as 487 km^2^, covering large parts of irrigation channels all over the country [36]. The resulting problematic link of aquatic vegetation, snail abundance and increased infection rates with *Schistosoma* spp. and *Fasciola* spp. was already recognized long ago and has been further studied since then, mainly with focus on vectors of schistosomiasis [37], [38]. Depending on focal outbreaks of diseases, the irrigation channels in Fayoum governorate are treated in loose intervals with molluscicides by the Ministry of Health, especially for prevention of schistosomiasis. In this context, water hyacinths will not only provide a habitat for *P. columella*, but might also be a refuge for the snail, when molluscicides are present in the water. Amphibious snails like P. *columella* might just move on the plants above water level and endure until the waves of molluscicides have passed. *P. columella* can also withstand detrimental circumstances by digging into moist mud where the snails survive even centimeters away from water [16]. Their ability to survive adverse conditions might be one reason for the success of this snail as an invader and explains the dominance of *P. columella* at most sampling sites in the present study.

We believe that the reported scenario of invasive *P. columella* as efficient snail host for *F. gigantica* represents a case of parasite spill-back, resulting in a drastic increase of infections in humans. This situation might be aggravated by the presence of water hyacinths, another invader that provides habitat for the snail intermediate host, altogether giving a good example how invasive species can alter biotic conditions and influence parasite life cycles, in this case of a human pathogen. A similar situation is likely to occur in many other agricultural areas in Africa.

Besides *F. gigantica*, *E. caproni* and an unidentified echinostome were also detected in the snails tested in the present study. To the best of our knowledge, this is the first report of *E. caproni* in *P. columella*. Echinostome cercariae are released from the first intermediate snail host, infect other snails and use them as second intermediate host where they form metacercariae. The respective final host becomes infected by ingestion of snails containing metacercariae. Detection of trematodes by molecular methods in the present study does not allow for distinction of sporocysts/rediae and metacercariae in the snails, but most likely *P. columella* is used as second intermediate host for *E. caproni* that normally infects *Biomphalaria* spp. as first intermediate hosts [39]. In cases of co-infections with *Schistosoma mansoni* and echinostome trematodes in the same snail, the latter were found to impair infection, development and infectivity of schistosome cercariae or even to consume larvae of other trematodes in the same snail (reviewed by Fried & Huffman and Loker & Adema [39], [40]). In case *P. columella* would prove to be first intermediate host for echinostomes, these parasites might have to be considered as regulating factor for *F. gigantica*. Therefore, the presence of echinostomes in *P. columella* in the Fayoum irrigation system might reduce the level of *F. gigantica*-infections in the area.

A recent study revealed the presence of different morphologically indistinguishable lineages *F. gigantica* from Africa and India that might in fact be two separate species, as well as an African highland lineage of *F. hepatica*-like flukes that use *Galba truncatula* as snail host and can be clearly differentiated genetically from European *F. hepatica* [41]. Various other studies have shown that species boundaries seem to be not clear for *F. hepatica* and *F. gigantica* (see review in Mas-Coma et al. [42]). Also, intermediate *Fasciola* individuals were described from Iran and Egypt, sharing characteristics of *F. hepatica* and *F. gigantica* [30], [43], [44]. The ITS1-5.8S-ITS2 rDNA sequences of *F. gigantica* from isolates obtained in the present study did not show the polymorphic sites characteristic for the intermediate forms reported by Amer et al. [44]. Therefore, we consider the infection in *P. columella* as “regular” *F. gigantica*. Nevertheless it is of great importance to clarify the identity of intermediate forms as well as lineages of *F. gigantica* and *F. hepatica*, their host spectrum and significance for human infections.

The large Fayoum oasis is a fertile agricultural area with a tight network of irrigation channels that provide ideal habitat for vector snails especially for *Schistosoma* and *Fasciola* spp. The invasive snail *P. columella* will contribute to the increased prevalence of fascioliasis in the human population in that area and put the population of about 2.9 million people (http://www.geohive.com/) at risk. Many of them live in agricultural areas and are exposed to parasitological problems linked to the irrigation system directly, but fascioliasis, in contrast to schistosomiasis, might even affect the urban populations, if crops bearing metacercariae are transported to the cities [1]. The present study showed that *F. gigantica* seems to be the major cause of fascioliasis in the Fayoum governorate and that the invasive snail *P. columella* is responsible for the maintenance of the infection in spite of snail eradication programs. Further studies are required to evaluate the effectiveness of molluscicide treatments on *P. columella* and to evaluate alternative approaches of snail control.

## References

[1] Mas-Coma S (2004) Chapter 19: Human fascioliasis. In: Cotruvo JA ,Dufour A ,Rees G ,Bartram J ,Carr R, et al.. (Eds.), World Health Organization (WHO), Waterborne Zoonoses: Identification, Causes and Control. IWA Publishing, London, pp. 305–322.

[2] Bargues MD, Mas-Coma S (2005) Reviewing lymnaeid vectors of fascioliasis by ribosomal DNA sequence analyses. J Helminthol 79: 257–267. 16153320 10.1079/joh2005297

[3] Bargues MD, Funatsu IR, Oviedo JA, Mas-Coma S (1996) Natural water, an additional source for human infection by *Fasciola hepatica* in the Northern Bolivian Altiplano. Parassitologia. 38(1–2): 251.

[4] El Sayed MH, Allam AF, Osman MM (1997) Prevention of human fascioliasis: a study on the role of acids, detergents and potassium permanganate in clearing salads from metacercariae. J Egypt Soc Parasitol 27(1): 163–169. 9097538

[5] Curtale F, Abd El-Wahab Hassanein Y, Wakeel A El, Mas-Coma S, Montresore A (2003) Distribution of human fascioliasis by age and gender among rural population in the Nile Delta, Egypt. J Trop Pediatr 49: 264–268. 14604157 10.1093/tropej/49.5.264

[6] Esteban JG, González C, Curtale F, Muñoz-Antoli C, Valero MA, et al (2003) Hyperendemic fascioliasis associated with schistosomiasis in villages in the Nile Delta of Egypt. Am J Trop Med Hyg 69(4): 429–437. 14640504

[7] Soliman MFM (2008) Epidemiological review of human and animal fascioliasis in Egypt. J Infect Dev Ctries 2: 182–189. 19738348 10.3855/jidc.260

[8] WHO (1995) Control of foodborne trematode infections. World Health Organ Tech Rep Ser 849: 1–157. 7740791

[9] Lotfy WM, El-Morshedy HN, El-Hoda MA, El-Tawila M, Omar E, et al (2002) Identification of the Egyptian species of *Fasciola* . Vet Parasitol 103: 323–332. 11777611 10.1016/s0304-4017(01)00613-6

[10] Dar Y, Amer S, Mercier a, Courtioux B, Dreyfuss G (2012) Molecular identification of *Fasciola* spp. (Digenea: Fasciolidae) in Egypt. Parasite 19: 177–182. 22550630 10.1051/parasite/2012192177 PMC3671433

[11] Mas-Coma S, Esteban JG, Bargues MD (1999) Epidemiology of human fascioliasis: a review and proposed new classification. Bull World Health Organ 77: 340–346. 10327713 PMC2557647

[12] Mas-Coma S, Bargues MD, Valero MA (2005) Fascioliasis and other plant-borne trematode zoonoses. Int J Parasitol 35: 1255–1278. 16150452 10.1016/j.ijpara.2005.07.010

[13] Farag HF, El Sayad MH (1995) *Biomphalaria alexandrina* naturally infected with *Fasciola gigantica* in Egypt. Trans R Soc Trop Med Hyg 89(1): 36. 7747303 10.1016/0035-9203(95)90648-7

[14] Ahmed AH, Ramzy RM (1999) Infection of two lymnaeid snails with *Fasciola gigantica* in Giza, a field study. J Egyptian Soc Parasitol 29: 687–696. 12561910

[15] Dar YD, Rondelaud D, Dreyfuss G (2005) Update of fasciolosis-transmitting snails in Egypt (review and comment). J Egypt Soc Parasitol 35: 477–490. 16083061

[16] van Eeden JA, Brown DS (1966) Colonization of fresh waters in the Republic of South Africa by *Lymnaea columella* Say (Mollusca: Gastropoda). Nature 210: 1172–1173. 5964184 10.1038/2101172a0

[17] de Kock KN De, Joubert PH, Pretorius SJ (1989) Geographical distribution and habitat preferences of the invader freshwater snail species *Lymnaea columella* (Mollusca: Gastropoda) in South Africa. Onderstepoort J Vet Res 56: 271–275. 2626264

[18] Madsen H, Frandsen F (1989) The spread of freshwater snails including those of medical and veterinary importance. Acta Trop 46: 139–146. 2566266 10.1016/0001-706x(89)90030-2

[19] Brown DS (1994) Freshwater snails of Africa and their medical importance. Taylor and Francis CRC Press, London, U.K. 608 p.

[20] de Kock KN, Wolmarans CT (2008) Invasive alien freshwater snail species in the Kruger National Park, South Africa. Koedoe 50 (1): 49–53.

[21] Kelly DW, Paterson RA, Townsend CR, Poulin R, Tompkins DM (2009) Parasite spillback: a neglected concept in invasion ecology? Ecology 90: 2047–2056. 19739367 10.1890/08-1085.1

[22] Sures B (2011) Parasites of animals. In: Simberloff D, Rejmánek M, (Eds.): Encyclopedia of Biological Invasions, 500–503, University of California Press, Berkely and Los Angeles.

[23] Abo-Madyan AA, Morsy TA, Motawea SM, Morsy ATA (2004) Clinical trial of Mirazid in treatment of human fascioliasis, Ezbet El-Bakly (Tamyia Center) Al-Fayoum Governorate. J Egypt Soc Parasitol 34: 807–818. 15587309

[24] Morsy TA, Salem HS, Haridy FM, Rifaat MMA, Abo-Zenadah NYA, et al (2005) Farm animals' fascioliasis in Ezbet El-Bakly (Tamyia Center) Al-Fayoum Governorate. J Egypt Soc Parasitol 35: 825–832. 16333892

[25] Folmer O, Black M, Hoeh W, Lutz R, Vrijenhoek R (1994) DNA primers for amplification of mitochondrial cytochrome c oxidase subunit I from diverse metazoan invertebrates. Mol Mar Biol Biotechnol 3: 294–299. 7881515

[26] Rózsa L, Reiczigel J, Majoros G (2000) Quantifying parasites in samples of hosts. J Parasitol 86(2): 228–232. 10780537 10.1645/0022-3395(2000)086[0228:QPISOH]2.0.CO;2

[27] R Core Team (2013) R: A language and environment for statistical computing. R Foundation for Statistical Computing, Vienna, Austria. Available: http://www.R-project.org/.

[28] Curtale F, Hammoud ES, El Wakeel A, Mas-Coma S, Savioli L (2000) Human fascioliasis, an emerging public health problem in the Nile Delta, Egypt. Res Rev Parasitol 60: 129–134.

[29] Marcilla A, Bargues MD, Mas-Coma S (2002) A PCR-RFLP assay for the distinction between *Fasciola hepatica* and *Fasciola gigantica* . Mol Cell Probes 16: 327–333. 12477436 10.1006/mcpr.2002.0429

[30] Periago MV, Valero M, El Sayed M, Ashrafi K, El Wakeel A, et al (2008) First phenotypic description of *Fasciola hepatica*/*Fasciola gigantica* intermediate forms from the human endemic area of the Nile Delta, Egypt. Infect Genet Evol 8: 51–58. 18006385 10.1016/j.meegid.2007.10.001

[31] Kendall SB, Parfitt JW (1965) The life-history of some vectors of *Fasciola gigantica* under laboratory conditions. Ann Trop Med Parasitol 59: 10–16. 14297347 10.1080/00034983.1965.11686275

[32] El-Shazly AM, Helmy MM, Haridy FM, El-Sharkawy EM, Morsy TA (2002) Fasciola immature stages sought in *Lymnaea* species and *Biomphalaria* species in the water bodies of Dakahlia Governorate. J Egypt Soc Parasitol 32(1): 109–118. 12049247

[33] Vázquez AA, Sánchez J, Pointier J-P, Théron A, Hurtrez-Boussès S (2013) *Fasciola hepatica* in Cuba: compatibility of different isolates with two intermediate snail hosts, *Galba cubensis* and *Pseudosuccinea columella* *J Helminthol* 1–7. doi:10.1017/S0022149X13000382 10.1017/S0022149X13000382 23721926

[34] Caron Y, Righi S, Lempereur L, Saegerman C, Losson B (2011) An optimized DNA extraction and multiplex PCR for the detection of *Fasciola* sp. in lymnaeid snails. Vet Parasitol 178: 93–99. 21242033 10.1016/j.vetpar.2010.12.020

[35] Gopal B (1987) Aquatic Plant Studies 1. Water hyacinth. Elsevier, Amsterdam, p. 484.

[36] Fayad YH, Ibrahim AA, El-Zoghby AA, Shalaby FF (2001) Ongoing activities in the biological control of water hyacinth in Egypt. In: Julien MH, Hill MP, Center TD, Jianquig D, (Eds.). Biological and integrated control of water hyacinth *Eichhornia crassipes* Proceedings of the 2^nd^ Meeting of the Global Working Group for the Biological and Integrated Control of Water Hyacinth, Beijing, China, 9–12 October 2000.

[37] Dawood IK, Farooq M, Dazo BC, Miguel LC, Unrau GO (1965) Herbicide trials in the snail habitats of the Egypt 49 project. Bull World Health Organ 32: 269–287. 14310915 PMC2555215

[38] Coles GC, Kabatereine NB (2008) Water hyacinth and the transmission of schistosomiasis. Trans R Soc Trop Med Hyg 102(6): 619–620 doi:10.1016/j.trstmh.2008.01.009 18374376 10.1016/j.trstmh.2008.01.009

[39] Fried B, Huffman JE (1996) The Biology of the intestinal trematode *Echinostoma caproni* . Adv Parasitol 38: 311–368. 8701798 10.1016/s0065-308x(08)60037-8

[40] Loker ES, Adema CM (1995) Schistosomes, echinostomes and snails: comparative immunobiology. Parasitol Today 11: 120–124.

[41] Walker SM, Prodöhl PA, Hoey EM, Fairweather I, Hanna REB, et al (2012) Substantial genetic divergence between morphologically indistinguishable populations of *Fasciola* suggests the possibility of cryptic speciation. Int J Parasitol 42: 1193–1199. 23131680 10.1016/j.ijpara.2012.10.007

[42] Mas-Coma S, Valero MA, Bargues MD (2009) Chapter 2. *Fasciola*, lymnaeids and human fascioliasis, with a global overview on disease transmission, epidemiology, evolutionary genetics, molecular epidemiology and control. Adv Parasitol 69: 41–146. 19622408 10.1016/S0065-308X(09)69002-3

[43] Ashrafi K, Valero MA, Panova M, Periago MV, Massoud, et al (2006) Phenotypic analysis of adults of *Fasciola hepatica*, *Fasciola gigantica* and intermediate forms from the endemic region of Gilan. Iran Parasitol Int 55: 249–260. 16901748 10.1016/j.parint.2006.06.003

[44] Amer S, Dar Y, Ichikawa M, Fukuda Y, Tada C, et al (2011) Identification of *Fasciola* species isolated from Egypt based on sequence analysis of genomic (ITS1 and ITS2) and mitochondrial (NDI and COI) gene markers. Parasitol Int 60: 5–12. 20888427 10.1016/j.parint.2010.09.003

